# Food insecurity, mental health and quality of life among people living with HIV commencing antiretroviral treatment in Ethiopia: a cross-sectional study

**DOI:** 10.1186/s12955-016-0440-8

**Published:** 2016-03-03

**Authors:** Markos Tesfaye, Pernille Kaestel, Mette Frahm Olsen, Tsinuel Girma, Daniel Yilma, Alemseged Abdissa, Christian Ritz, Martin Prince, Henrik Friis, Charlotte Hanlon

**Affiliations:** Department of Psychiatry, College of Health Sciences, Jimma University, Jimma, Ethiopia; Department of Nutrition, Exercise and Sports, Faculty of Science, University of Copenhagen, Copenhagen, Denmark; Department of Pediatrics and Child Health, College of Health Sciences, Jimma University, Jimma, Ethiopia; Department of Internal Medicine, College of Health Sciences, Jimma University, Jimma, Ethiopia; Department of Medical Laboratory Sciences & Pathology, College of Health Sciences, Jimma University, Jimma, Ethiopia; Centre for Global Mental Health, Institute of Psychiatry, King’s College London, London, UK; Department of Psychiatry, School of Medicine, College of Health Sciences, Addis Ababa University, Addis Ababa, Ethiopia

**Keywords:** Food insecurity, Common mental disorder, Quality of life, PLHIV, Africa

## Abstract

**Background:**

Studies from high-income settings show that both food insecurity and common mental disorders (CMDs) are associated with lower quality of life among people living with HIV (PLHIV). However, there is limited research among PLHIV in sub-Saharan Africa. In this study we tested the hypothesis that food insecurity and CMDs would be associated with poorer quality of life of PLHIV in Ethiopia.

**Methods:**

A cross-sectional study was carried out with 348 PLHIV who were initiating antiretroviral therapy recruited from two primary care centers and a tertiary Hospital in southwest Ethiopia. Food insecurity, CMD, and quality of life were measured using instruments adapted and validated in Ethiopia (Household Food Insecurity Access Scale, Kessler-6, and WHOQOL-HIV-BREF-ETH, respectively). Multiple linear regression analysis was used to identify factors associated with quality of life after adjusting for confounders.

**Results:**

The prevalence of severe household food insecurity among PLHIV was 38.7 %. After adjusting for confounders, severe food insecurity (β = -3.24, 95 % CI: -6.19; -0.29) and higher levels of CMD symptoms (β = -1.72 for each 1 point increase, 95 % CI: -1.94; -1.49) were associated with lower quality of life. Other factors associated with lower quality of life were advanced HIV disease (β = -3.80, 95 % CI: -6.18; -1.42), and being underweight (BMI = 17.0 – 18.5 kg/m^2^) (β = -3.45, 95 % CI: -6.18; -0.71). Owning more household assets was associated with higher quality of life (β = 0.99 for owning one more asset, 95 % CI: 0.09; 1.89).

**Conclusion:**

Poor mental health and food insecurity are associated with lower quality of life in PLHIV. There is a need for longitudinal studies to elucidate the pathways linking CMD, food insecurity and quality of life.

## Background

Food insecurity, defined as “the limited or uncertain availability of nutritionally adequate and safe foods, or limited or uncertain ability to acquire acceptable foods in socially acceptable ways”, is a global problem [1]. In 2009, the Ethiopian national nutrition report estimated that food insecurity affected 35 % of the population [2]. The common mental disorders (CMD), depression and anxiety, are major contributors to the global burden of disease, with depression accounting for 9.6 % of disability [3]. Both food insecurity and CMD symptoms have been found to be increased in people living with HIV (PLHIV) and associated with poorer HIV outcomes.

### Food insecurity in people living with HIV

Prevalence estimates of all forms of food insecurity among PLHIV in sub-Saharan Africa (SSA) were 63 % in Ethiopia [4], 75 % in Uganda [5], 57 % in the Democratic Republic of Congo [6], and 52 % in Tanzania [7]. In line with this, severe food insecurity was common among PLHIV in SSA, ranging from 38 to 66 % [5, 8]. A bidirectional relationship has been hypothesized between HIV and food insecurity, with food insecurity associated with increased risk of HIV infection, as well as HIV infection leading to food insecurity [9]. In addition to its nutritional consequences, food insecurity has been linked to poorer health outcomes, including incomplete viral suppression [10, 11], lower CD4^+^ count [11] and poor adherence to antiretroviral therapy (ART) [6, 12, 13].

### CMD in people living with HIV

Studies indicate that CMDs are common among PLHIV and lead to worse medical outcomes. A meta-analysis has shown PLHIV to have between two and five times higher prevalence of major depressive disorder than HIV negative controls [14]. In a systematic review, the average point prevalence of depressive disorders and anxiety disorders among PLHIV was found to be 41 and 34 %, respectively, in low and middle-income countries [15]. A study of CMDs among PLHIV receiving ongoing care in three public hospitals in Ethiopia found that approximately half of them had probable CMD [16]. That figure is much higher than the estimate for the general population in Ethiopia, which is 17 % [17]. In PLHIV, CMDs, especially depression, are associated with a decrease in CD4 T-lymphocytes, increase in viral load and elevated risk of mortality [18] as well as lower quality of life [19, 20]. The existing evidence indicates an association between CMDs and non-adherence to ART [21]. On the other hand, CMDs have been associated with risky sexual behaviors that lead to HIV infection [22].

### Interrelationship between food insecurity, CMD and quality of life

**Fig. 1:**
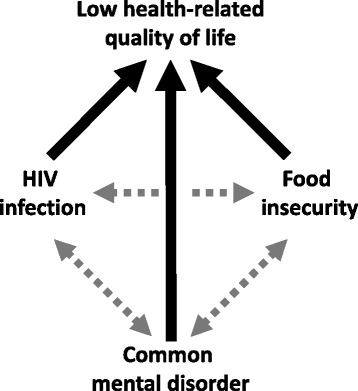
The interrelationship among food insecurity, HIV, CMDs, and Quality of life

There appears to be complex interrelationships between food insecurity, CMDs and quality of life in PLHIV (Fig. 1). In a systematic review of studies from low-income countries, food insecurity was found to be associated with CMDs [23]. In studies carried out in general population samples in Ethiopia, food insecurity has also been found to be associated with higher levels of CMD symptoms [24, 25]. Similarly, studies done in PLHIV in low-income countries have found food insecurity was associated with CMDs, although only in women [5, 26]. Evidence that food insecurity leads to higher CMDs rather than vice versa comes from a longitudinal study among poor communities within the US where food insecure PLHIV were found to become depressed [27].

While access to adequate food and nutrition in day-to-day life has been highlighted as an important dimension of quality of life among people living in low-income settings [28], studies have also found that food insecurity contributes to lower quality of life [29, 30]. In SSA, there is a lack of data on the independent effect of food insecurity on the quality of life of PLHIV. A study on a community sample of ART-naïve PLHIV from Uganda found that severe food insecurity was associated with lower quality of life independent of dietary quality [8]. However, the study did not assess the nutritional and the mental health status of the PLHIV and their relative contribution to lower quality of life.

CMDs have also been linked to lower quality of life among PLHIV. Although studies from Ethiopia, and India reported CMDs to be associated with lower quality of life among PLHIV, these studies did not consider the potential confounding effect of food insecurity [19, 20, 31].

Improving quality of life is an important goal for HIV treatment programmes, but the existing evidence base is limited by the absence of studies examining the relative contributions food insecurity and CMD. In this study, we sought to investigate whether food insecurity and CMDs are associated with lowered quality of life in PLHIV, after adjusting for socio-economic status and malnutrition. The findings will help guide research and interventions aimed at improving the quality of life of PLHIV in low-income settings.

## Methods

### Study design and setting

A cross-sectional study was conducted in Jimma zone (sub-region), southwest Ethiopia between July 2010 and August 2012. The study was nested within a randomized controlled trial of a lipid-based nutrient supplement given to PLHIV who were initiating ART [32]. The sites included the ART clinics of Jimma University Specialized Hospital (JUSH), Jimma Health Centre and Agaro Health Centre.

### Participants and sampling

A consecutive sample of 348 PLHIV who were eligible to start ART at the study sites were invited to take part in the study. Exclusion criteria for this study were age <18 years, pregnancy, lactation, diabetes mellitus and current use of nutritional supplements.

### Data collection procedures

Data were collected by clinical nurses, who were trained on the data collection procedures. The clinicians working in the ART clinic referred all ART eligible adult PLHIV to the research team. The data were collected just before ART initiation. The clinical nurses provided information regarding the study and evaluated the eligibility of participants for the study. Eligible participants who gave written consent to participate in the study underwent interviews conducted by the clinical nurses. Weight and height were measured to compute body mass index (BMI). Blood samples were collected from the participants for laboratory investigations such as hemoglobin and CD_4_ cell count.

### Main outcome measure

#### Quality of life

The “bref” version of the World Health Organization Quality of Life –HIV module (WHOQOL-HIV) was adapted and validated for use among Ethiopian PLHIV in Amharic and Afaan Oromo languages. The original tool has been validated to ensure semantic, item and measurement equivalences, and construct validity was found. (Tesfaye M, Olsen MF, Medhin G, Friis H, Hanlon C, Holm L: Adaptation and validation of the short version WHOQOL-HIV in Ethiopia, submitted). The adapted instrument, namely WHOQOL-HIV-BREF-ETH, has 27-items asking the respondents to rate the different aspects of their subjective experience of their living standards in the 2 weeks before the interview. The responses were scored from 1 to 5. Lower scores indicate worse quality of life. The total scores on the WHOQOL-HIV-BREF-ETH which included two general items and six domains were used in the multivariable regression analysis. The total quality of life scores could range from a minimum value of 26 and a maximum of 130.

### Main exposures

#### Food insecurity

This was assessed using the nine item version of the Household Food Insecurity Access Scale (HFIAS) with questions asking whether the respondent has experienced any of the indicators of food insecurity in the previous 1 month period [33]. This scale has previously been validated in Ethiopia [34].

#### Common mental disorders (CMD)

The Kessler-6 scale (K6) [35], which is a shorter version of the Kessler-10 scale, was used to measure CMDs. K6 is composed of six questions asking the respondents how often they have experienced symptoms of CMDs in the previous 30 days. The responses are coded on a Likert-scale from zero indicating ‘never’ to 4 indicating ‘all of the time’. The K6 has been validated for detection of CMDs in the Ethiopian setting [36].

### Potential confounders

Socio-demographic characteristics: age, sex, and marital status of the participants were recorded on structured questionnaires.

Socio-economic characteristics: highest level of education achieved, occupation, assets owned, and floor material of the houses were recorded using a structured and pre-tested questionnaire which was developed by the investigators.

BMI: height (in centimeters) and weight (in kilograms) of the participants were measured using a stadiometer and calibrated weighing scales, respectively.

WHO stage: the severity of HIV disease was graded using the World Health Organization (WHO) clinical staging with stage I indicates asymptomatic HIV whereas stage IV indicates the advanced disease.

### Data management and analyses

Data were double entered using EpiData version 3.1 (EpiData Association, Odense, Denmark) and analysed with STATA IC version 11 (StataCorp LP, College Station, TX). Data on quality of life were grouped into domains (physical, independence, psychological, social, environment, and spirituality), and the mean values for the domains were calculated as recommended by the WHOQOL-HIV Group [37]. Food security was categorized as follows: food secure if none of the items were endorsed, mild food insecurity if the respondent endorsed any of the items 1, 2, 3, and/or 4 but not the items 5 to 9, ‘moderate food insecurity’ if the respondent has endorsed items 5 and/or 6 but not the items 7 to 9, and ‘severe food insecurity’ the respondent has endorsed items 7, 8 and/or 9 as previously used by other investigators [25]. Mild food insecurity represented reduction in the quality of food while moderate and severe food insecurity represented quantitative reduction.

Background characteristics of the PLHIV were presented as means (SD) and percentages. Bivariate and multiple linear regression analyses were conducted to test the associations of CMD symptoms and food insecurity with the total scores on the WHOQOL-HIV-BREF-ETH among PLHIV. Each potential confounder, i.e. socio-demographic characteristics, education, marital status, WHO stage, and nutritional status, was added to the model sequentially. As the data was collected over a period of 2 years, the effect of season i.e. dry and rainy on the model fit was also analyzed. The variables were only included in the multiple linear regression if they were found to significantly improve the model fit using the likelihood ratio test. The association between food insecurity and CMD symptoms was tested using multiple linear regression.

## Results

### Sample characteristics

As reported previously [38], out of 453 PLHIV recruited, a total of 348 (76.8 %) were enrolled. The mean age of the participants was 32.9 (±8.8) years. The majority (66.7 %) were women, and more than half (52.9 %) were either divorced or widowed. Moderate and severe food insecurity were both common with a prevalence of 67.2 % (Table 1).

**Table 1:** Background characteristics of 348 adults with HIV

	*N* (%)
Age, mean (SD)	32.9 (8.8)
Sex	
Male	116 (33.3)
Female	232 (66.7)
Marital status	
Married	129 (37.3)
Divorced or widowed	183 (52.9)
Never married	32 (9.3)
Occupation	
Employed	78 (22.4)
Daily labour/local trade	119 (34.2)
Housewife	65 (18.7)
Unemployed	42 (12.1)
Other	44 (12.6)
Education	
No formal education	101 (29.0)
Primary	179 (50.3)
Secondary and above	72 (20.7)
Assets owned	
< 2 assets	169 (48.6)
≥ 2 assets	179 (51.4)
Floor material	
Earth/sand/dung	251 (74.5)
Tiles/cement/parquet	86 (25.5)
Food insecurity	
None	45 (13.1)
Mild	68 (19.8)
Moderate	98 (28.5)
Severe	133 (38.7)
Body mass index [Kg/m^2^]	
Severely low (<16)	30 (8.7)
Moderately low (16 - 17)	32 (9.2)
Underweight (17 – 18.5)	95 (27.4)
Normal (18.5 – 25)	181 (52.2)
Overweight (>25)	9 (2.6)

The mean hemoglobin (SD) of the PLHIV was 128 (20.2) g/L. The WHO clinical stage of the PLHIV showed that 29.2 % (100) had stage I disease, 29.8 % (102) had stage II, and 32.5 % (111) had stage III, while only 8.5 % (29) of the PLHIV had stage IV disease. The mean CD4 count was 187 (110) cells/uL, and 20.8 % of the PLHIV had at least one opportunistic infection.

### Food insecurity, CMDs and total quality of life scores in PLHIV

As shown in Table 2, increasing severity of food insecurity was associated with lower quality of life (test-for-trend, *p* = 0.009). A higher level of CMD symptoms was associated with lower quality of life (β = -1.72, 95 % CI: -1.94; -1.49). Furthermore, having advanced HIV disease (β = -3.80, 95 % CI: -6.18; -1.42), and having mild malnutrition (BMI = 17.0–18.5 Kg/m^2^) were also associated with lower quality of life scores (β = -3.45, 95 % CI: -6.18; -0.71). In the adjusted model, moderate to severely low BMI was not associated with lower quality of life (β = -0.70, 95 % CI: -3.87; 2.46). However, the coefficients for mildly low BMI and that for moderate to severely low BMI were not significantly different from each other (*P* = 0.12). On the other hand, the number of household assets owned was positively associated with higher quality of life (β =0.99, 95 % CI: 0.09; 1.89). Season was not associated with quality of life scores.

**Table 2:** Factors associated with total quality of life in a sample of 348 adults with HIV

Characteristic	Unadjusted β (95 % CI)	Adjusted β (95 % CI)^a^
Sex		
Male	Reference	Reference
Female	−1.35 (-4.64; 1.94)	−0.01 (-2.84; 2.83)
Age, years	−0.05 (-0.23; 0.12)	−0.11 (-0.25; 0.04)
Marital status		
Married	Reference	Reference
Widowed/divorced	−5.78 (-9.06; -2.49)	−0.57 (-3.18; 2.04)
Never married	−4.21 (-9.89; 1.46)	−1.63 (-5.95; 2.68)
Educational level		
No formal education	Reference	Reference
Primary	5.89 (2.31; 9.47)	1.00 (-1.82; 3.82)
Secondary and above	6.95 (2.50; 11.40)	0.73 (-2.98; 4.45)
Household assets (out of 7)	2.01 (0.94; 3.07)	0.99 (0.09; 1.89)
Food insecurity		
None or Mild	Reference	Reference ^b^
Moderate	−7.62 (-11.45; -3.78)	−2.52 (-5.55; 0.51)
Severe	−10.06 (-13.61; -6.51)	−3.24 (-6.19; -0.29)
Body mass index [Kg/m^2^]		
Normal or overweight (≥18.5)	Reference	Reference
Mildly low (17-18.5)	−5.46 (-9.07; -1.85)	−3.45 (-6.18; -0.71)
Moderate to severely low (<17.0)	−4.69 (-8.88; -0.50)	−0.70 (-3.87; 2.46)
CMD scores	−1.84 (-2.06; -1.62)	−1.72 (-1.94; -1.49)
WHO stage of HIV		
Stage I & II	Reference	Reference
Stage III & IV	−5.26 (-8.42; -2.10)	−3.80 (-6.18; -1.42)
Season		
Dry	Reference	
Rainy	−0.32 (-3.54; 2.91)	-

^a^Adjusted for age, sex, marital status, education, household assets, food insecurity, BMI, WHO stage, and CMD scores

^b^ Test for trend was significant (*p* = 0.009)

### Food insecurity and CMDs

As shown in Table 3, a higher burden of CMD symptoms was observed with increasing severity of food insecurity. Severe food insecurity (β = 2.40, 95 % CI: 1.05; 3.74) had a significantly stronger association with CMD symptoms than moderate food insecurity (β = 2.31, 95 % CI: 0.92; 3.71), (*p* = 0.001). Moreover, CMD symptoms were negatively associated with primary education (β = -1.66, 95 % CI: -2.99; -0.35), and positively associated with moderate and severe malnutrition i.e. BMI less than 17 kg/m^2^ (β = 1.62, 95 % CI: 0.16; 3.07) as well as with being widowed or divorced (β = 0.96, 95 % CI: 0.25; 2.18).

**Table 3:** Factors associated with common mental disorders in a sample of 348 adults with HIV

Characteristic	Unadjusted β (95 % CI)	Adjusted β (95 % CI)^a^
Sex		
Male	Reference	Reference
Female	1.44 (0.28; 2.61)	0.85 (-0.49; 2.18)
Age, years	−0.03 (-0.09; 0.03)	−0.02 (-0.09; 0.05)
Marital status		
Married	Reference	Reference
Widowed/divorced	1.86 (0.69; 3.04)	0.96 (0.25; 2.18)
Never married	0.62 (-1.39; 2.63)	−0.40 (-2.44; 1.65)
Educational level		
No formal education	Reference	Reference
Primary	−2.26 (-3.54; -0.99)	−1.66 (-2.99; -0.35)
Secondary and above	−2.09 (-3.66; -0.52)	−0.96 (-2.60; 0.69)
Household assets (out of 7)	−0.34 (-0.73; 0.04)	-
Food insecurity		
None or Mild	Reference	Reference
Moderate	2.52 (1.13; 3.91)	2.31 (0.92; 3.71)
Severe	2.89 (1.61; 4.18)	2.40 (1.05; 3.74)
Body mass index [Kg/m^2^]		
Normal or overweight (≥18.5)	Reference	Reference
Mildly low (17-18.5)	0.90 (-0.40; 2.20)	0.99 (-0.28; 2.26)
Moderate to severely low (<17.0)	1.73 (0.22; 3.24)	1.62 (0.16; 3.07)
WHO stage of HIV		
Stage I & II	Reference	
Stage III & IV	0.33 (-0.81; 1.47)	-

^a^Adjusted for age, sex, marital status, education, food insecurity, and BMI

## Discussion

This study found that two-thirds of PLHIV initiating ART lived in households affected by moderate to severe food insecurity. Food insecurity and higher levels of CMD symptoms were associated with lower quality of life. Also, advanced HIV disease and lower BMI were associated with lower quality of life. PLHIV who owned household assets appear to have better quality of life. However, age, sex, and marital status were not found to be associated with quality of life among PLHIV. Furthermore, food insecurity was associated with a high burden of CMD symptoms.

The proportion of PLHIV who reported moderate and severe household food insecurity is comparable to a previous report from the same setting [4] and to other reports from SSA [6]. Household food insecurity among PLHIV in our sample was nearly double that of the general population in Ethiopia [2]. This suggests that PLHIV are vulnerable to food insecurity. Accordingly, a study done in Canada has found that food insecurity was five times more prevalent among PLHIV than the general population [39]. It has been indicated that PLHIV in particular could suffer from food insecurity resulting from the decreased productivity, and increased healthcare expenditures [9].

In line with previous evidence [8, 29], the findings provide further evidence on the association of food insecurity with quality of life. In low-income settings, the significance of access to adequate food and nutrition for a person’s quality of life has been highlighted previously [28]. It is likely that individuals from food insecure households also experience general deprivation and poverty. This is supported by our findings that ownership of household assets being associated with higher quality of life. Thus, food support programs as well as financial support might lead to improved quality of life among PLHIV. The apparent attenuation of the association between lower quality of life and moderately and severely low BMI after adjusting for confounders might be due to underpowered sample size. Thus, PLHIV who exhibit undernutrition may have lower quality of life as reported by another study from India [40].

Previous studies have found CMDs to be associated with poor quality of life among PLHIV [19, 20, 31]. Our data also suggest that CMDs may have interaction with food insecurity, although the direction of association cannot be determined in this cross-sectional study. Prospective studies are required in order to further elucidate the direction of association between CMDs and quality of life. Policies and program guidelines aimed at improving the quality of life of PLHIV who are starting ART treatment need to address the mental healthcare needs of this population in addition to tackling food insecurity.

People with chronic illnesses generally report poorer quality of life than their healthy counterparts. Our finding on the association between stage of HIV disease and quality of life is consistent with other studies that have demonstrated that PLHIV who are symptomatic have poorer quality of life than healthy individuals and even asymptomatic PLHIV [40]. It is possible that the quality of life of PLHIV who are effectively treated with ART may not be different from that of people who are HIV negative. Indeed, a recent study from Uganda has found that PLHIV on ART had better quality of life than a comparable community sample [41]. Accordingly, it may be the general health condition, and the fear and the stigma of the PLHIV, which may partly resolve with effective treatment with ART, rather than HIV serostatus per se that accounted for the poorer quality of life in this study.

Food insecurity has been linked to poorer mental health in a systematic review of studies from low-income countries [23] as well as in community studies from high-income countries [42–44]. Similarly, studies on PLHIV have also found association between food insecurity and poorer mental health [41, 45, 46]. The observed relationship between more severe food insecurity and poorer mental health in our study supports a causal association although this cannot be confirmed due to the cross-sectional nature of the analysis. In Ethiopia, adults suffering from depression have been found to experience functional disability [47] and hence loss of financial resources. The available evidence supports that treatment of CMDs generally results in improved income of the patients and their families [48]. It is possible that the household food insecurity in our study sample reflected the extreme levels of poverty which may have acted as a chronic stressor leading to a high burden of CMD symptoms. By contrast, the number of household assets owned, a measure of socio-economic status, was not associated with mental health. The absence of assets is not an immediate stressor and thus, may not be expected to have an association with mental health status, whereas food insecurity represents acute poverty and is felt immediately as a stressor.

In addition to food insecurity, low level of education was one of the measures of poverty that was found to be consistently associated with CMDs in a systematic review [49]. Our data also supports the previous findings from low-income settings. The attenuation of the association between a secondary and higher education and CMD symptoms might be due to the smaller sample size of that particular group. However, the exact mechanism as to how lower education might lead to higher burden of CMD symptoms needs further research.

The link between BMI and poor mental health is inconsistent in the literature. Studies have found CMDs to be associated with obesity [49–52], and low BMI [53, 54]. Other studies did not find any association between BMI and CMDs [55, 56]. The observed association between low BMI and CMD symptoms indicates that undernourished PLHIV have increased mental healthcare needs in addition to nutritional and medical interventions.

Our findings may not be generalized to all PLHIV as participants were recruited from health facilities and they represent a segment of the community with better access to health services. The PLHIV who did not participate may be systematically different from those who participated in this study, and the results might have been biased as a result. The assessment of CMDs was not based on gold standard clinical interview but rather using the Kessler scale which is a screening tool. Nevertheless, evidence supports that persons with greater number of CMD symptoms have higher likelihood of being a case of CMD, and experiencing disability. Both food insecurity and CMDs have previously been linked to poor physical health status. Social support which might buffer the association between food security, and mental health and quality of life could have confounded the results.

## Conclusion

Poor mental health and food insecurity are associated with lower quality of life in PLHIV. There is a need for longitudinal studies to elucidate the pathways linking CMD, food insecurity and quality of life.

### Ethics approval and consent to participate

The study was approved by the Ethiopian National Health Research Ethics Review Committee and the Research Ethical Review Committee of Jimma University. Written informed consent was sought from participants of the study.

### Availability of data and materials

Additional data are available on request from the corresponding author.

## Abbreviations

ART: antiretroviral therapy
BMI: body mass index
CMD: common mental disorder
HFIAS: household food insecurity access scale
JUSH: Jimma University Specialized Hospital
K6: Kessler-6 scale
PLHIV: people living with HIV
SSA: Sub-Saharan Africa
WHO: World Health Organization
WHOQOL-HIV: World Health Organization quality of life –HIV module
